# Evaluation of Varying Ductile Fracture Criteria for 42CrMo Steel by Compressions at Different Temperatures and Strain Rates

**DOI:** 10.1155/2014/579328

**Published:** 2014-01-30

**Authors:** Guo-zheng Quan, Gui-chang Luo, An Mao, Jian-ting Liang, Dong-sen Wu

**Affiliations:** School of Material Science and Engineering, Chongqing University, Chongqing 400044, China

## Abstract

Fracturing by ductile damage occurs quite naturally in metal forming processes, and ductile fracture of strain-softening alloy, here 42CrMo steel, cannot be evaluated through simple procedures such as tension testing. Under these circumstances, it is very significant and economical to find a way to evaluate the ductile fracture criteria (DFC) and identify the relationships between damage evolution and deformation conditions. Under the guidance of the Cockcroft-Latham fracture criteria, an innovative approach involving hot compression tests, numerical simulations, and mathematic computations provides mutual support to evaluate ductile damage cumulating process and DFC diagram along with deformation conditions, which has not been expounded by Cockcroft and Latham. The results show that the maximum damage value appears in the region of upsetting drum, while the minimal value appears in the middle region. Furthermore, DFC of 42CrMo steel at temperature range of 1123~1348 K and strain rate of 0.01~10 s^−1^ are not constant but change in a range of 0.160~0.226; thus, they have been defined as varying ductile fracture criteria (VDFC) and characterized by a function of temperature and strain rate. In bulk forming operations, VDFC help technicians to choose suitable process parameters and avoid the occurrence of fracture.

## 1. Introduction

42CrMo (American grade: AISI 4140) is one of the representative medium carbon and low alloy steel. Due to its good balance of strength, toughness, and wear resistance, 42CrMo high-strength steel is widely used for many general purpose parts including automotive crankshaft, rams, spindles, crow bars, and ring gears. 42CrMo steel contains chromium and molybdenum as alloying elements and may be heat treated over a wide range to give the combined advantages of proper hardness, strength, and ductility [1]. An important concern in the plastic forming processes of high-strength steel is whether the desired deformation can be performed without any fracture of the material. Fracturing by ductile damage occurs quite naturally in metal forming processes due to the development of microcracks associated with large straining or due to plastic instabilities associated with material behavior and boundary conditions. In industrial practice, however, the empirical know-how of the designer is decisive for the fracture-free quality of the products, but it often requires intensive efforts and tremendous time [2]. Therefore, there is a critical need for predicting and preventing fracture, which is a major feature of the forming processes and the quality of the products. If it is possible to predict the conditions within the deformed workpiece which lead to fracture, then it may be feasible to choose appropriate process parameters conditions and to modify the forming processes to produce sound and reliable products, with obviously savings in time and cost [3, 4].

As a vital index of formability, the ductile damage in plastic forming process can be described as a function of local temperature, strain, and the stress state. The resulting damage can be calculated, for example, with the model of effective stresses, considering crack closure effects by splitting the Cauchy stress tensor into a compressive and tensile part. Therefore, the materials damage state in industrial processes can be simulated by implementing damage models into FE software. Thus, the damage degree, that is, the fracture tendency, can be characterized as the ration of damage value and ductile fracture criteria (DFC). Historically, ductile fracture criteria are based on experimental work that utilizes a deformation process that is related to actual industrial applications [5]. However, such approaches are time consuming and rarely lead to a general solution of future defect problem. So far, the fracture criterion such as Cockcroft-Latham is suited for tenacity fracture in bulk metal forming simulation [6]. Usually the critical damage value is considered as a constant of material like yield stress, stress limit. Cockcroft and Latham [2–9] have not expounded whether the critical damage value depends on the temperature and strain rate. But in metal forming processes, deformation condition varies drastically in a different region and a different forming stage, so it is necessary to set up a fracture criterion applicable for various deformation conditions.

The object of this study is to investigate the natural relationships between damage evolution and deforming parameters of 42CrMo high-strength steel and then to construct ductile fracture criteria histogram along with deforming parameters. An innovative concept of damage sensitive rate was brought forth as the essential intermediate quantity to evaluate ductile fracture criteria. As for the innovative approach, heat compression simulations, finite element simulations, and mathematic computations are necessary, which induces that physical experiments and numerical computations provide mutual support to determine the varying ductile fracture criteria (VDFC). Without a doubt, based on the VDFC, the fracture position and moment of 42CrMo high-strength steel during various forming processes can be predicted accurately and conveniently.

## 2. Experimental Procedure

The 42CrMo high-strength steel employed in the present investigation was provided in the form of bar with the diameter of 100 mm. Its chemical compositions (wt. %) are as follows: C-0.450, Si-0.280, Cr-0.960, Mn-0.630, Mo-0.190, P-0.016, Cu-0.014, S-0.012, and Fe (balance). Before the experiment, the homogenized ingot was scalped to diameter 10 mm and height 12 mm with grooves on both sides filled with machine oil mingled with graphite powder as lubricant to reduce friction between the anvils and specimen, and sixteen of such specimens were machined with their cylinder axes parallel to the axial line direction of the bar. A computer-controlled, servohydraulic Gleeble 1500 testing machine was used for the compression testing. It can be programmed to simulate both the thermal and the mechanical industrial process variables for a wide range of hot deformation conditions. The specimens were resistance heated at a heating rate of 1 K/s by thermocouple feedback-controlled AC current and held at a certain temperature for 180 s before compression tests, which decreased the anisotropy in flow deformation behavior effectively. Three thermocouples were welded at the midspan of billet to provide accurate temperature control and measurement during testing. Then the compression tests corresponding to a height reduction ratio of 60% were carried out at four different temperatures of 1123 K, 1198 K, 1273 K and 1348 K and four different strain rates of 0.01 s^−1^, 0.1 s^−1^, 1 s^−1^ and 10 s^−1^.

During the compressing process, the variations of stress and strain were monitored continuously by a personal computer equipped with an automatic data acquisition system. The true stress-strain relationships were derived from the measurement of the nominal stress-strain curves collected according to the following formula: *σ*_*T*_ = *σ*_*N*_(1 + *ε*_*N*_), *ε*_*T*_ = ln⁡(1 + *ε*_*N*_), where *σ*_*T*_ is the true stress, *σ*_*N*_ the nominal stress, *ε*_*T*_ the true strain, and *ε*_*N*_ the nominal strain [10].

## 3. Flow Stress Behavior

**Figure 1:**
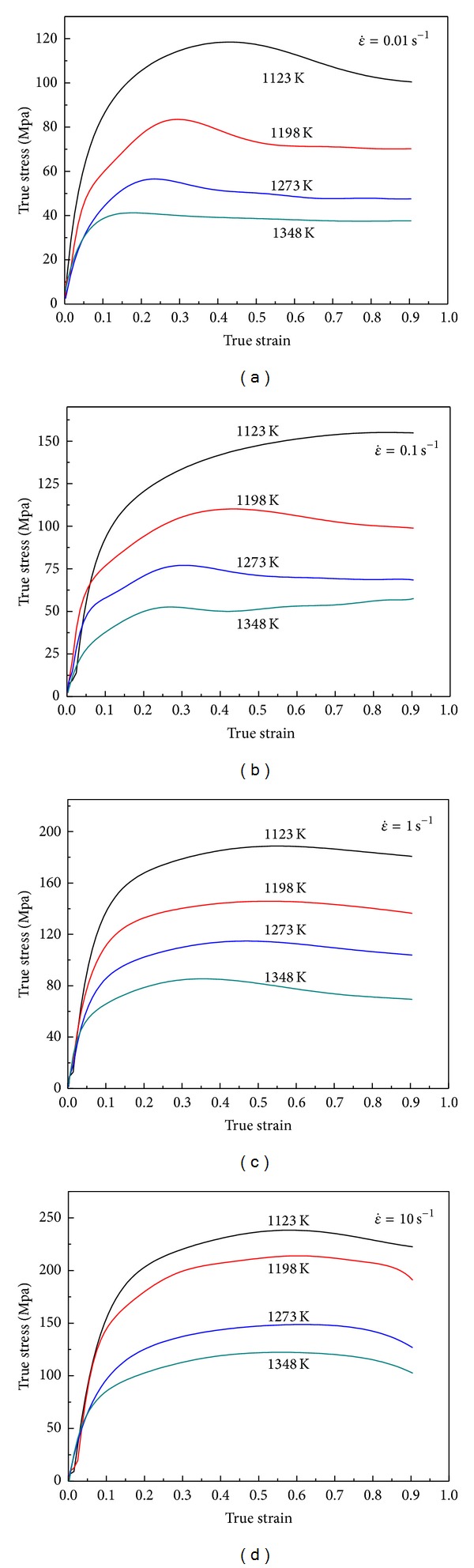
The true stress-strain curves of 42CrMo steel under the different deformation temperatures with strain rates (a) 0.01 s^−1^, (b) 0.1 s^−1^, (c) 1 s^−1^, and (d) 10 s^−1^.

The true compressive stress-strain curves obtained from the hot compression tests of 42CrMo steel are depicted in Figures 1(a)–1(d). It can be found that the temperature and strain rate on the flow stress are significant. Comparing these curves with one another, it is found that, for a specific strain rate, the flow stress decreases markedly with increasing temperature. In contrast, for a fixed temperature, the flow stress generally increases as the strain rate increases. The cause lies in the fact that lower strain rate and higher temperature provide longer time for the energy accumulation and higher mobilities at boundaries which result in the nucleation and growth of dynamically recrystallized grains and dislocation annihilation. From the true stress-strain curves in Figures 1(a)–1(d), it can be seen that the stress evolution with strain exhibits three distinct stages. At the first stage where work hardening (WH) predominates, flow stress exhibits a rapid increase to a critical value. At the second stage, flow stress exhibits a smaller and smaller increase until reaching a peak value or an inflection of work hardening rate, which shows that the thermal softening due to DRX and dynamic recovery (DRV) becomes more and more predominant, and then it exceeds WH. At the third stage, three types of curve variation tendency can be generalized as following: decreasing gradually to a steady state with DRX softening (1123–1348 K and 0.01 s^−1^, 1198–1348 K and 0.1 s^−1^, and 1273–1348 K and 1 s^−1^), maintaining higher stress level without significant softening and work-hardening (1123–1198 K and 1 s^−1^, 1123–1348 K and 10 s^−1^), and increasing continuously with significant work-hardening (1123 K and 0.1 s^−1^). Thus, it can be concluded that the typical form of flow curve with DRX softening, including a single peak followed by a steady state flow as a plateau, is more recognizable at high temperatures and low strain rates. That is because at higher strain rates and lower temperatures, the higher work-hardening rate slows down the rate of DRX softening, and both the peak stress and the onset of steady state flow are therefore shifted to higher strain levels.

## 4. Damage Computation

### 4.1. Cockcroft-Latham's DFC

The workability of metal plays a major role for judging whether the metal will be manufactured successfully or caused by ductile fracture in the forming process. And the ductile fracture is usually the main reason for the failed workpiece. So the prediction of the fracture initiation is an important item in the forming category. Based on various hypotheses, many criteria for ductile fracture have been proposed empirically as well as theoretically [11, 12]. It is well known that the forming limit of metals depends greatly upon the deformation history. Therefore, the history of stress and strain may have to be considered in the criteria. Based on cumulative damage theory, Cockcroft and Latham [9, 11–15] have proposed a damage computation module allowing for a critical value of the tensile strain energy per unit of volume, which has been applied successfully to a variety of loading situations. It has been used to predict fracture in such processes as extrusion, rolling, and upsetting. Cockcroft-Latham's damage [9, 12, 13] can be expressed as an amount of work that the ratio of maximum tensile stress *σ*_*T*_ to effective stress $\overset{-}{\sigma }$ carries out through the applied equivalent strain $\overset{-}{\varepsilon }$ in a metal-working process; that is,

(1) $$\begin{array}{c} D=\int_{0}^{{\overset{-}{\varepsilon }}_{f}}\frac{\sigma_{T}}{\overset{-}{\sigma }}\text{d}\overset{-}{\varepsilon }, \end{array}$$

where ${\overset{-}{\varepsilon }}_{f}$ is the total equivalent strain corresponding to fracture failure, and the maximum damage value, *D*_max⁡_, is named ductile fracture criteria (DFC), that is the critical damage value of the workpiece to fracture. The criterion is not based on a micromechanical model to fracture, but simply recognizes the dependence of the critical value at fracture upon the level of the largest principal stress. The DFC value, *D*_max⁡_, is dependent on the same material parameters that forming limits are dependent on. Material metallurgical properties such as the microstructure, grain form, grain size and the inclusion content of non-metallic, have a significant effect on the critical damage value. The magnitude of *D* cannot exceed *D*_max⁡_ to failure, if the value of (1), *D*, reaches *D*_max⁡_, fracture should occur in material at the integration point taken under consideration. Thus, by comparing *D* with *D*_max⁡_, the risk of material failure during processing can be assessed.

In order to calculate *D* by FE simulation, (1) has to be converted to an appropriate discrete expression that convenient for FE code; that is,

(2) $$\begin{array}{c} D=\int_{0}^{{\overset{-}{\varepsilon }}_{f}}\frac{\sigma_{T}}{\overset{-}{\sigma }}\frac{\text{d}\overset{-}{\varepsilon }}{\text{d}t}\text{d}t=\int_{0}^{t_{f}}\frac{\sigma_{T}}{\overset{-}{\sigma }}\dot{\overset{-}{\varepsilon }}\text{d}t≅\sum_{0}^{t_{f}}\frac{\sigma_{T}\dot{\overset{-}{\varepsilon }}\Delta t}{\overset{-}{\sigma }}, \end{array}$$

where $\dot{\overset{-}{\varepsilon }}$ is the equivalent strain rate, *t*_*f*_ is the total time corresponding to fracture failure, and Δ*t* is the variable time increment used in the FE analysis.

### 4.2. Approach to Determine Cockcroft-Latham Type DFC

From the true stress-strain curves of 42CrMo steel, it can be found that 42CrMo high-strength steel is a typical strain-softening alloy. As is well known, for strain-hardening alloy the fracture criteria can be determined by directly comparing the simulation with the destructive experiments in terms of the critical deformation level. However, for strain-softening alloy the ductile fracture criteria cannot be determined directly because it is difficult to find visible cracks on the surface of deformed billet and also the stress-strain curves do not have apparent fracture points. Thus, it is necessary to find an indirect way to evaluate the ductile damage criteria for strain-softening alloy. However, it is a nontrivial issue that still needs to be addressed in greater depth. An innovative indirect (nondestructive) method to evaluate DFC has been brought forward in this research. A basic research approach that physical experiments, numerical simulations and mathematic computations provide mutual support for the critical damage factor was established. As several series of billet samples had been compressed on a heat physical simulation machine under different deformation temperatures and strain rates, the true stress-strain data collected resulted in the performance of simulations through an integration method using the subroutine in the FE software of DEFORM-2D.

According to cumulative damage theory, the damage value a moment ago is less than that a moment later during a compressing process. Therefore, the maximum value seems to appear at the last compressing step, but it does not mean fracture step. As the cumulation characters maybe contribute to find the critical damage value, it is necessary to simulate and analyze the damage cumulating process. For this reason, an innovative concept about the sensitive rate of Cockcroft-Latham damage (as (3)) in plastic deformation (*R*_step_) is brought out and defined as the ratio of the damage increment at one step (Δ*D*) to the accumulated value (*D*_acc_). It is supposed that if the maximum damage value will keep increasing in a very small growth rate near to zero, it means that fractures have appeared [9]. Thus, the fracture time, that is, fracture strain or fracture height reduction can be identified and determined. Consider

(3) $$\begin{array}{c} R_{\text{step}}=\frac{\Delta D}{D_{\text{acc}}}. \end{array}$$

**Figure 2:**
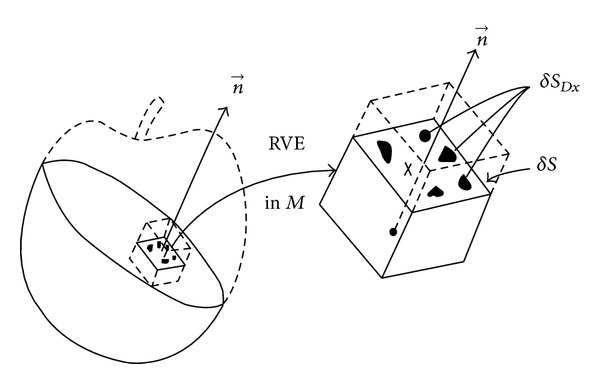
Damaged RVE in a damaged body.

The emergence of the assumptions above responds to Kachanov's [16–18] explanation of damage. Kachanov explained the “one-dimensional surface damage variable” by considering a damaged body and a representative volume element (RVE) at a point *M* oriented by a plane defined by its normal $\vec{n}$ and its abscissa *x* along the direction $\vec{n}$ (as shown in Figure 2) [16]. The value of the damage $D(M,\vec{n},x)$ attached to the point *M* in the direction $\vec{n}$ and the abscissa *x* is

(4) $$\begin{array}{c} D\left(M,\vec{n},x\right)=\frac{\delta S_{Dx}}{\delta S}, \end{array}$$

where *S*_*Dx*_ is the area of intersection of all the flaws with the plane defined by the normal $\vec{n}$ and abscissa *x*; *S* is the total area at the intersection plane.

Damage *D* is bounded as 0 ≤ *D* ≤ *D*_*c*_, where *D*_*c*_ is a critical damage value corresponding to the decohesion of atoms. *D* = 0 represents the undamaged RVE material, and *D* = *D*_*c*_ represents the rupture failure in the remaining resisting area. As rupture failure has occurred, damage *D* will keep increasing in a growth rate near to zero or equal to zero with increasing deformation strain. It means that the damage will not be sensitive to deformation.

## 5. Computation Results

### 5.1. Ductile Damage Cumulating Process

**Figure 3:**
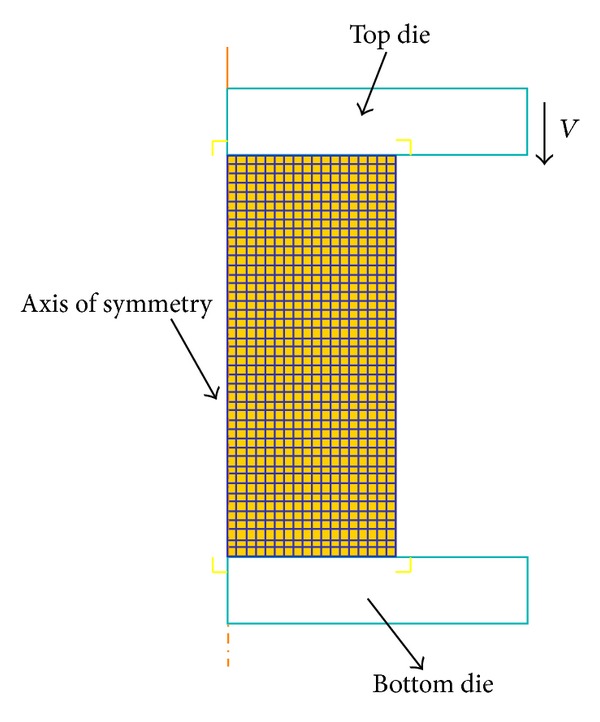
The basic FE model of compression testing in DEFORM-2D.

**Figure 4:**
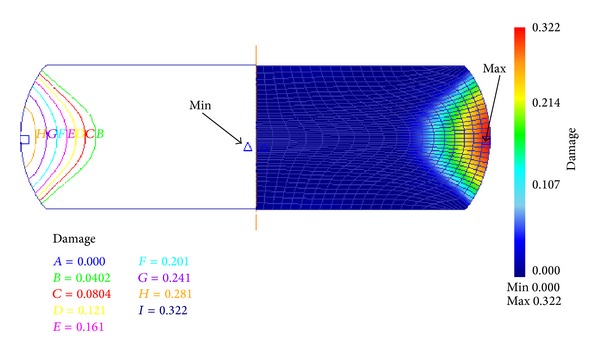
The damage distribution of the height reduction of 60% at temperature 1348 K and strain rate 0.01 s^−1^.

Rigid-plastic FE models were established in the DEFORM-2D platform to simulate the above corresponding upsetting tests. The basic model to represent compression testing method is shown as in Figure 3. Because of symmetry, in order to simplify and reduce the running time during the simulation, only one-half of the geometry is considered. The specimen was modeled as a plastic object; the tools were modeled as rigid surfaces. The initial mesh of billet consists of 792 four node elements; both sides of the cylinder workpiece because are filled with machine oil mingled with graphite powder as lubrication in the hot compression test, the frictional between the specimen and the tools is assumed to be 0.1. For the simulation of plastic deformation process, the material model for billet can be defined by inputting the true stress-strain curve date (as Figure 1). And then the damage value during the deformation process was calculated.  Figure 4 shows the damage distribution of the height reduction of 60% at temperature of 1348 K and strain rate of 0.01 s^−1^. From the simulation results of all the isothermal hot compression tests at the temperatures of 1123 K, 1198 K, 1273 K, and 1348 K, the strain rates of 0.01 s^−1^, 0.1 s^−1^, 1 s^−1^, and 10 s^−1^, it can be easily found that the maximum damage value always appears in the region of upsetting drum corresponding to higher strain, stress, and strain rate, while the minimal value appears in the middle region. As the result shown in Figure 4, the most severely damaged material is located in the upsetting drum, the damage extent decreasing sharply in diameter from outside to inside. Therefore, the crack initialization will necessarily take place at the edge of the sample.

**Figure 5:**
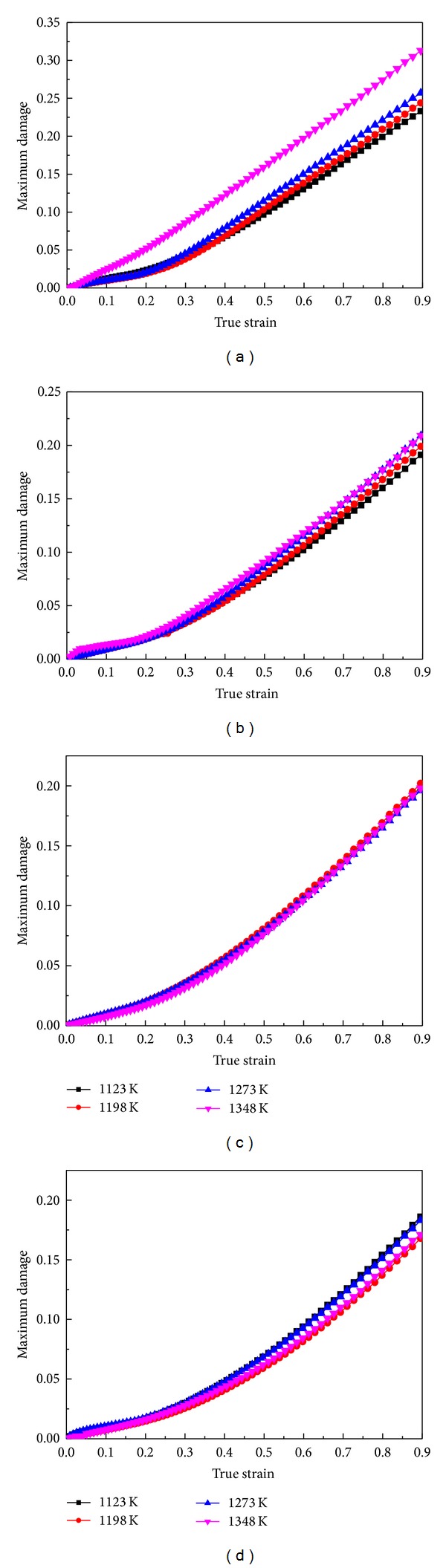
The maximum damage varying during compressing process at different temperatures and strain rates: (a) 0.01 s^−1^, 1123~1348 K; (b) 0.1 s^−1^, 1123~1348 K; (c) 1 s^−1^, 1123~1348 K; (d) 10 s^−1^, 1123~1348 K.

**Figure 6:**
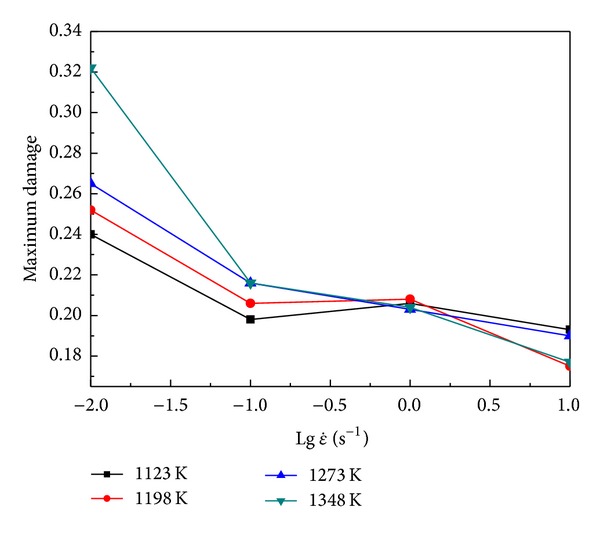
The relationships between maximum damage and strain rate at the end of compressing process under different temperatures.

Based on the simulation results, the incremental ratios of Cockcroft-Latham type damage during the whole upsetting processes at different temperatures and strain rates were calculated and shown in Figures 5(a)–5(d). It is obvious that the damage value increases nonlinearly as the compressive true strain increases from 0 to about 0.4, and then it increases nearly linearly to the end. Comparing these curves with one another, it is found that, as the strain rates are 0.01 s^−1^ and 0.1 s^−1^, for a fixed true strain, the maximum cumulated damage increases with increasing temperature, while there is no obvious regular for the strain rates of 1 s^−1^ and 10 s^−1^. In contrast, as the temperatures are 1123 K and 1198 K, with the strain rate increases, in the beginning, the maximum cumulated damage decreases rapidly, and then has slight increasement; at last, the damage value decreased again. When the temperatures are 1273 K and 1348 K, the damage value decreases with increasing strain rate (as shown in Figure 6). Further, it can be summarized that the changes in strain rate have a more significant effect on the cumulated damage; that is to say, the damage cumulating process is more sensitive to strain rate.

### 5.2. Evaluation of Varying Ductile Fracture Criteria (VDFC)

**Figure 7:**
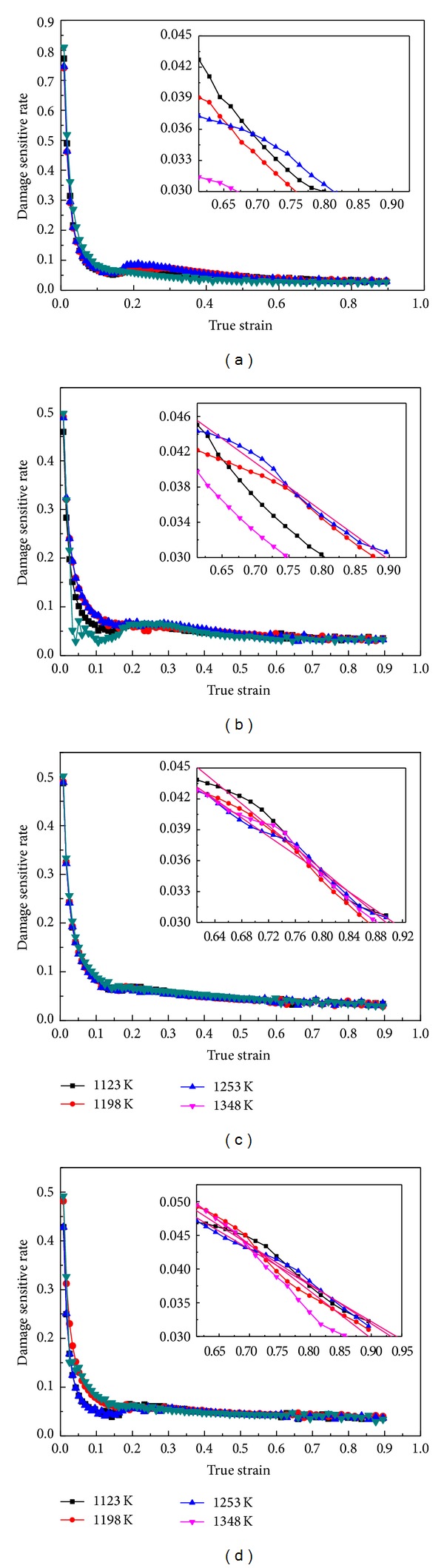
The variation of damage sensitive rate under different temperatures and strain rates: (a) 0.01 s^−1^, (b) 0.1 s^−1^, (c) 1 s^−1^, and (d) 10 s^−1^.

In order to evaluate the ductile fracture criteria, the fracture occurrence and the damage value at fracture time are necessary to be made certain. Based on the damage cumulating processes as shown in Figure 5, the sensitive rates (*R*_step_) of Cockcroft-Latham damage (as (3)) at different temperatures and strain rates were evaluated and shown in Figures 7(a)–7(d). It can be seen that, for all these deformation conditions, *R*_step_ decreases to the trough point rapidly before true strain 0.05~0.1; then it has a slight increase in true strain 0.1~0.2, soon after which it decreases to zero by slow degrees in true strain range of 0.2~0.92. In this study, the zero point has been added a tolerance 0~0.03, and it is assumed as the fracture time in tolerance limits. That is to say, when *R*_step_ decreases to 0.03, it means that fracture has appeared. To find the fracture time, the point arrays after true strain 0.6 were picked out from each incremental ratio varying curve and fitted linearly. The intersection of line fitted and horizontal axis is obtained, and it is made certain as the fracture step.

**Figure 8:**
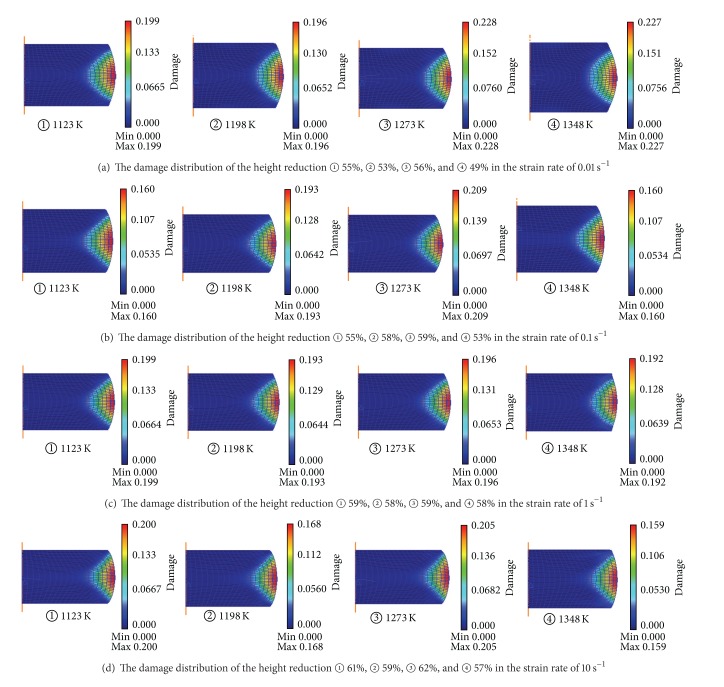
The fracture height reduction and the correspondent DFC under different temperatures and strain rates: (a) 0.01 s^−1^, 1123~1348 K; (b) 0.1 s^−1^, 1123~1348 K; (c) 1 s^−1^, 1123~1348 K; (d) 10 s^−1^, 1123~1348 K.

**Figure 9:**
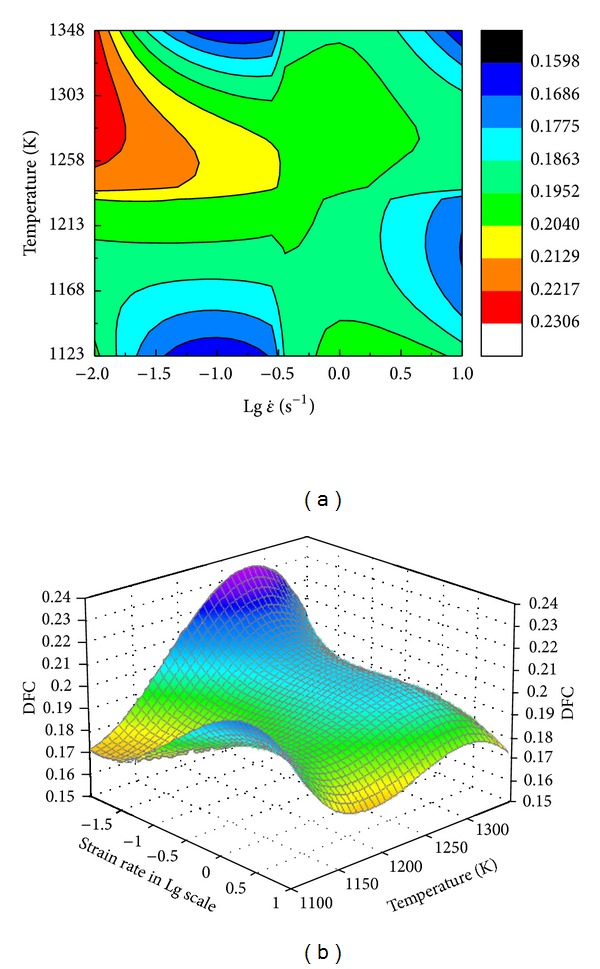
The temperature and strain rate effect on the ductile fracture criteria of 42CrMo steel: (a) contour plot, (b) response surface.

As the fracture time has been determined, based on Figure 5, the fracture analysis step and the correspondent DFC under different deformation conditions were evaluated as in Figure 8. Obviously, under different temperatures and strain rates, DFC of 42CrMo steel are not constant, which are affected by the working condition. Figures 9(a)-9(b) show the contour plot and response surface of DFC; they indicate the natural relationships between deforming conditions and DFC of 42CrMo high-strength steel. As can be seen in Figure 9, the ductile fracture criteria (DFC) are not constant but change in a range of 0.16~0.226; thus, they can be defined as varying ductile fracture criteria (VDFC) and described as a function of strain rate and temperature (as Table 1). In the scatter diagram, it is found that there is a ridgeline under the deformation condition of temperature which is about 1275 K, and DFC value decreases with increasing strain rate, on the both sides of the ridgeline DFC value decreases gradually; as the strain rate is 0.01~0.063 s^−1^ and temperature range is 1250~1300 K, there is a peak value, what is worth explaining is that the forming process will be better under these conditions due to the higher DFC. Therefore, it can be educed that cracks will appear more difficulty under the deformation conditions 1250~1300 K and 0.01~0.063 s^−1^ and cracks will appear most easily under the deformation conditions 1198 K & 10 s^−1^ due to the lowest DFC. It is very significant to guide the practical production.

## 6. Conclusions

Based on the criteria proposed by Cockcroft and Latham, the ductile damage cumulating process, fracture initiation sites and DFC diagram for 42CrMo steel along with various deformation conditions were evaluated. The following conclusions were obtained

1. In a typical strain-softening material of 42CrMo steel, the maximum damage value appears in the region of upsetting drum, while the minimal value appears in the middle region. The crack initialization will necessarily take place at the edge of the workpiece.
2. DFC of 42CrMo steel in the temperature range of 1123~1348 K and the strain rate range of 0.01~10 s^−1^ are not a constant but changes with a range of 0.16~0.227, thus they have been defined as varying ductile fracture criteria (VDFC) and characterized by a function of temperature and strain rate.
3. Based on the VDFC diagram, the exact fracture moment during various forming processes can be predicted effectively and conveniently. In addition, the deformation conditions with lower fracture risk of 42CrMo steel have been identified as 1250~1300 K & 0.01~0.063 s^−1^.

## Figures and Tables

**Table 1:** An approximate analytical formula for the VDFC of 42CrMo steel.

VDFC	Exponents	Remarks
*z* = *a* + *bx* + *cy* + *dx* ^2^ + *ey* ^2^ + *fxy* + *gx* ^3^ + *hy* ^3^ + *ixy* ^2^ + *jx* ^2^ *y*	*a* = 34.178246	Error = 0.0058031112
	*b* = 1.5461689	
	*c* = −0.08381634	
	*d* = −0.057663468	
	*e* = 6.8770317*e* − 05	
	*f* = −0.002485447	
	*g* = −0.0073097269	
	*h* = −1.8770657*e* − 08	
	*i* = 9.9622534*e* − 07	
	*j* = 3.8651909*e* − 05	
